# Polyhexanide and chlorhexidine loaded chitosan wound dressings

**DOI:** 10.1080/07853890.2021.1896112

**Published:** 2021-09-28

**Authors:** Eleni Nydrioti, Benilde Saramago, Ana Paula Serro

**Affiliations:** a Centro de Química Estrutural, Instituto Superior Técnico, University of Lisbon, Lisbon, Portugal; b Centro de Investigação Interdisciplinar Egas Moniz (CiiEM), Egas Moniz Cooperativa de Ensino Superior, Caparica, Portugal

## Abstract

**Introduction:**

Wounds may be caused by surgery, trauma, or as a result of diseases such as diabetes. The disruption of the skin/internal tissues and the contact with the external environment may lead to microbial infection. Wound dressings are capable of providing a protective barrier and accelerate the wound healing process [1]. Chitosan is one of the most promising materials for wound dressings, due to its good biocompatibility, low toxicity, haemostatic properties, antibacterial activity and biodegradability [2]. The main goal of this work is to produce chitosan-based hydrogels to be used as efficient and safe drug delivery platforms.

**Materials and methods:**

Two different chitosan-based materials were produced starting from Bioceramed formulations, AbsorKi (suitable for absorbing exudate from the wound) and HemoKi (enhancing haemostasis). The latter was modified by the addition of genipin, a cross-linking agent. The dressings were individually loaded with two different drugs, polyhexamethylene biguanide (PHMB, also called polyhexanide) and chlorhexidine diacetate (CHX), by soaking in solutions containing each drug (24 h, 5 mL, 36 °C, 180 rpm, 0.5 mg/mL for PHMB and 5 mg/mL for CHX). The physical properties (swelling, tensile strength) of the materials were studied prior and upon sterilisation by high hydrostatic pressure (HHP, 600 MPa, 10 min, 70 °C). Surface morphology was analysed by scanning electron microscopy (SEM). *In vitro* drug release studies were performed with a Franz diffusion cell system, combined with UV–Vis absorption spectroscopy for drug quantification. The efficiency of HHP was evaluated by sterility tests. Chorioallantoic membrane (HET-CAM) tests were done to study potential irritation of the skin.

**Results:**

Both dressings present a porous structure and an extremely high swelling capacity (>1700%) before sterilisation. HPP affected the materials in different ways: it increased the swelling capacity of AbsorKi but decreased it for HemoKi. The drug release profiles indicated that the concentrations of PHMB and CHX increased in a sustained way on the first day. HHP increases the amount of drug released, except for AbsorKi with PHMB. Regarding the mechanical properties, sterilisation improved the resistance of both dressings. HET-CAM tests suggest that the produced materials do not lead to irritation.

**Discussion and conclusions:**

HHP revealed an efficient method to ensure the materials sterilisation making the drug loaded wound dressings potentially efficient devices for the absorption of exudate from the wound bed. The combination of chitosan, a natural antibacterial agent, with the studied disinfectant and antiseptic drugs may lead to promising materials to be used as drug delivery platforms. Further studies in animal models are needed to conclude about the safety and clinical usefulness of the developed dressings.
